# High or Nonhigh Doses of Proton Pump Inhibitors for Patients with Peptic Ulcer Bleeding?

**DOI:** 10.1155/2013/803139

**Published:** 2013-02-18

**Authors:** Yu-Hsi Hsieh, Hwai-Jeng Lin

**Affiliations:** ^1^Division of Gastroenterology, Department of Medicine, Buddhist Dalin Tzu Chi General Hospital, No. 2, Min-Sheng Road, Dalin, Chia-Yi 622, Taiwan; ^2^School of Medicine, Buddhist Tzu Chi University, No. 701, Zhongyang Rd., Sec. 3, Hualien 97004, Taiwan; ^3^Division of Gastroenterology and Hepatology, Department of Internal Medicine, Taipei Medical University Hospital, No. 252, Wuxing Street, Taipei 11031, Taiwan; ^4^School of Medicine, Taipei Medical University, Taipei, Taiwan

I read with interest the article entitled “A real world report on intravenous high-dose and nonhigh-dose proton pump inhibitors therapy in patients with endoscopically treated high-risk peptic ulcer bleeding” [1]. In this study, Lu et al. retrospectively analyzed patients receiving nonhighdose (80 mg pantoprazole i.v. bolus followed by i.v. 80 mg per day for 3 days) and high-dose proton pump inhibitors (PPI, 80 mg pantoprazole i.v. bolus followed by 8 mg per hr for 3 days) after obtaining initial hemostasis. After performing case-control matching, they found no statistical difference between the high-dose and nonhigh-dose groups. Therefore, they suggest that both doses of PPI were similar in reducing rebleeding in high-risk patients after successful endoscopic therapy. 

This conclusion is different from that in the consensus conference and also in our study [2, 3]. There are several key points that deserve to be mentioned with regards to this study. Lu's analysis is a retrospective study. Therefore, some important clinical variables could not be adjusted evenly between both groups. As a practice, doctors tend to use a high-dose PPI in high-risk patients after obtaining initial hemostasis. This point is demonstrated in Lu's study, Table 3. The number of patients with shock is more in the high-dose PPI group than that in the nonhigh-dose group (61.4% versus 46%). 

In Lu's study, the rebleeding rate for the high-dose group (19/70, 27.1%) is much higher than our series (2/50, 4%) and another report (8/120, 6.7%) [2, 4]. This phenomenon may be explained by the high percentage of patients with renal impairment (35/70, 50%). The high proportion of enrolled patients with renal impairment is unusual as compared to the past reports. Because three days after endoscopic therapy are a critical period, high-dose PPI is needed for these three days. After three days, patients usually receive oral intake. However, in Lu's study, they still gave 80 mg i.v. per day after three days. Thus, utilizing such therapy may waste some economic resources. 

In recent few years, there have been some articles supporting the use of low-dose PPI in high-risk patients after endoscopic hemostasis [5]. Many of these articles have pitfalls related to study design, such as the inclusion of patients with low-risk stigmata and the injection of epinephrine alone [6]. In vitro studies revealed that the acid environment impairs platelet function and clot stabilization [7]. Therefore, elevation of intragastric pH is mandatory to prevent rebleeding in patients with peptic ulcer bleeding, which has been confirmed in the consensus conference [2]. In our previous study, we obtained a markedly low rebleeding rate (4%) with a high-dose IV PPI [3]. Further, we found that different IV doses of PPIs have different rebleeding rates (omeprazole 160 mg/day: 9%, 6/67; 80 mg/day: 21.2%, 14/66) [8]. 

Clearly, there is a bit of a grey zone in identifying stigmata of recent hemorrhage (SRH) [9]. Misinterpretation of SRH can occur for a number of reasons, such as doctors' experience and academic judgement. Therefore, one strict design (double blind study) is favored in such a clinical trial. 
